# Domestic violence in Tunisian women during pregnancy and anxiety: which association?

**DOI:** 10.1192/j.eurpsy.2024.429

**Published:** 2024-08-27

**Authors:** R. Jbir, L. Aribi, I. Chaari, S. Mohamed, G. Fatma, M. Ines, N. Messedi, J. Aloulou

**Affiliations:** psychiatry B, Hedi chaker hospital university, Sfax, Tunisia

## Abstract

**Introduction:**

Domestic violence is a major public health problem. The situation is alarming in Arab countries: the prevalence of domestic violence is 39.3% in Saudi Arabia, 55% in Morocco and 62.2% in Egypt.

In Tunisia, a national survey carried out by the national family planning office in 2010, published in July 2011, drew attention for the first time to the frequency of this phenomenon in Tunisia and the recurrent nature of this form of violence. Unfortunately, few studies have focused on domestic violence during pregnancy and its impact on the mental health of expectant mothers.

**Objectives:**

To study the prevalence of domestic violence during pregnancy among Tunisian women consulting in the context of medical expertise and its association with anxiety.

**Methods:**

Our study was descriptive and analytical cross-sectional, carried out with women examined in the context of medical expertise following domestic violence at ‘Hedi Chaker hospital’,Sfax , from May 2021 until January 2022.

An anonymous survey was asked to these ladies, it included a section for collecting socio-demographic data.

The HADS questionnaire was used to screen for anxiety.

**Results:**

122 responses was collected. The average age of victims was 35.66 **±** 9.94 years.

All the women in our population study were married, and each one was a victim of at least one form of violence. The majority (86.1%) had children. Most of them had secondary (44.3%) or university (31.1%) level education.

More than half of the women (63.9%) had no occupation.

Sixty-five women (53.3%) were assaulted during pregnancy, 43% of whom suffered from complications of varying severity.

Different consequences on pregnancy were reported with decreasing prevalence: 16.9% hospitalization in a gynecological ward, 13.8% abortion, 6.2% fetal death in utero and premature delivery in 4.6% of cases.

According to the HADS, seventy-six of women surveyed (62.3%) had anxiety symptoms.

Anxiety was significantly associated with exposure to violence during pregnancy (p=0.03).

**Conclusions:**

Our results showed a significant incidence of domestic violence during pregnancy and a significant association with anxiety.

Different actions must be taken towards these anxious women such as: Identify a “referent” in maternity wards to screen for domestic violence and directing women to structures and shelters that can help and, above all, protect them.

**Disclosure of Interest:**

None Declared

